# Reference-Guided De Novo Genome Assembly to Dissect a QTL Region for Submergence Tolerance Derived from Ciherang-Sub1

**DOI:** 10.3390/plants10122740

**Published:** 2021-12-13

**Authors:** Yuya Liang, Shichen Wang, Chersty L. Harper, Nithya K. Subramanian, Rodante E. Tabien, Charles D. Johnson, Julia Bailey-Serres, Endang M. Septiningsih

**Affiliations:** 1 Department of Soil and Crop Sciences, Texas A&M University, College Station, TX 77843, USA; liangy27@msu.edu (Y.L.); nithya@tamu.edu (N.K.S.); 2 Genomics and Bioinformatics Service, Texas A&M AgriLife Research, College Station, TX 77843, USA; shichen.wang@gmail.com (S.W.); charlie@ag.tamu.edu (C.D.J.); 3 Texas A&M AgriLife Research Center, Beaumont, TX 77713, USA; c-harper@aesrg.tamu.edu (C.L.H.); RETabien@ag.tamu.edu (R.E.T.); 4 Center for Plant Cell Biology, Department of Botany and Plant Sciences, University of California Riverside, Riverside, CA 92521, USA; serres@ucr.edu

**Keywords:** submergence, Ciherang-Sub1, genome assembly, genome, rice

## Abstract

Global climate change has increased the number of severe flooding events that affect agriculture, including rice production in the U.S. and internationally. Heavy rainfall can cause rice plants to be completely submerged, which can significantly affect grain yield or completely destroy the plants. Recently, a major effect submergence tolerance QTL during the vegetative stage, *qSub8.1*, which originated from Ciherang-Sub1, was identified in a mapping population derived from a cross between Ciherang-Sub1 and IR10F365. Ciherang-Sub1 was, in turn, derived from a cross between Ciherang and IR64-Sub1. Here, we characterize the *qSub8.1* region by analyzing the sequence information of Ciherang-Sub1 and its two parents (Ciherang and IR64-Sub1) and compare the whole genome profile of these varieties with the Nipponbare and Minghui 63 (MH63) reference genomes. The three rice varieties were sequenced with 150 bp pair-end whole-genome shotgun sequencing (Illumina HiSeq4000), followed by performing the Trimmomatic-SOAPdenovo2-MUMmer3 pipeline for genome assembly, resulting in approximate genome sizes of 354.4, 343.7, and 344.7 Mb, with N50 values of 25.1, 25.4, and 26.1 kb, respectively. The results showed that the Ciherang-Sub1 genome is composed of 59–63% Ciherang, 22–24% of IR64-Sub1, and 15–17% of unknown sources. The genome profile revealed a more detailed genomic composition than previous marker-assisted breeding and showed that the *qSub8.1* region is mostly from Ciherang, with some introgressed segments from IR64-Sub1 and currently unknown source(s).

## 1. Introduction

Rice (*Oryza sativa* L.) is a staple food that feeds nearly half of the world’s population [1]. Complete submergence of rice reduces the availability of carbon dioxide and oxygen, reducing photosynthesis and limiting the aerobic metabolism, which together can dramatically reduce crop productivity [2]. In the U.S., for example, hurricane Harvey brought heavy rain and flooding that caused USD 7.5 million in losses for rice and soybean in Texas in 2017 [3]. Due to climate change, extreme weather events have increased the frequency of severe flooding, which significantly affects agriculture, including rice production in the U.S. and globally. A major submergence tolerance locus, *Submergence 1* (*Sub1*) from the landrace FR13A, was identified and introgressed into several popular rice varieties [4,5,6,7,8,9,10,11,12]. The key gene of this locus is *Sub1A-1*, encoding an ethylene-responsive factor (ERF) in subgroup VII, which is activated by ethylene that accumulates in submerged tissues [13]. The linked *Sub1B* and *Sub1C* genes encode the most related ERFs in the genome, indicating this locus arose through tandem duplication [14] prior to domestication [15]. 

Complete submergence during the vegetative stage is a polygenic trait. Efforts to identify additional resources for submergence tolerance include germplasm screening [16,17,18], quantitative trait loci (QTL) identification [19,20,21], and assessment of allelic sequence variation of *Sub1A* and the related *Sub1B* and *Sub1C* within the *Sub1* locus [14,15,22]. One of such efforts mapped a major effect submergence tolerance QTL (*qSub8.1*) on chromosome 8 using recombinant inbred lines derived from a cross between Ciherang-Sub1 and IR10F365 [20]. Both parents possessed the *Sub1A-1* gene, which provides about two weeks of tolerance to complete submergence during the vegetative growth phase [4,13]. The *qSub8.1* tolerant allele was derived from Ciherang-Sub1, and it accounts for about 28% of the phenotypic variance, with a heritability of 23.3% [20].

The International Rice Research Institute (IRRI) has worked closely with its national partners to introgress *Sub1A-1*, a submergence tolerance gene contributing the largest effect identified to date, into several popular rice varieties. There were six Sub1 lines developed in the first stage: Swarna-Sub1, Samba Mahsuri-Sub1, IR64-Sub1, TDK1-Sub1, CR1009-Sub1, and BR11-Sub1 [5,6,10]. Another two Sub1 lines (Ciherang-Sub1 and PSB Rc18-Sub1) were developed in the second stage [8]. In addition to providing tolerance to submergence, this gene also enhanced survival of rapid dehydration following desubmergence and water deficit under drought [23]. Previous reports also showed that Sub1 lines were more tolerant to leaf blast and bacterial blight under normal or submerged conditions [24,25]. The level of tolerance of these eight Sub1-lines under submergence stress is affected by genetic backgrounds and environments. Gene-to-gene interactions, including epistasis, may play a role in this case [26], and additional QTL regions may also contribute to the phenotypic variations. However, generally, we could still see the clear differences between the Sub1 lines and their original susceptible parents [5,6,8]. Additionally, thus far, there were no yield penalties reported with the introgression of Sub1 alone or Sub1 combined with other major effect QTL associated with drought tolerance or anaerobic germination [8,27,28]. Many of these lines have been released in various countries, especially in South Asia and South East Asia [7,27].

One of the parents used to map *qSub8.1* was Ciherang-Sub1, which was developed from a cross between Ciherang, a popular *indica* variety from Indonesia [8], and IR64-Sub1 [6] using just one backcross and one selfing generation (derived from a BC_1_F_2_ individual plant). After being completely submerged for 25 days, Ciherang-Sub1 had a survival rate of 89.9% compared to a survival rate of 3.7% for Ciherang. Ciherang-Sub1 also had a higher survival rate compared to other Sub1-introgressed rice varieties such as Swarna-Sub1 (77.9%), BR11-Sub1 (79.0%), PSB Rc18-Sub (58.2), and SambaMahsuri-Sub1 (65.8%) [8]. This improved variety has been released in several countries and has also been used in various genetic and molecular studies as an elite cultivar [7,20,28,29,30,31]. The second parent used to map *qSub8.1* was IR10F365, which is an advanced breeding line with *Sub1A* introgressed through conventional breeding methods. It was hypothesized that *qSub8.1* might have a complementary effect with *Sub1A* in the event of submergence during the vegetative stage [20]. A variety with prolonged submergence tolerance, beyond what *Sub1A* provides alone, would be beneficial to maintain rice production when we face the increasing effects of climate change. The objectives of the present study are to (a) characterize the region of *qSub8.1* by analyzing sequence information of the three associated cultivars (Ciherang-Sub1, Ciherang, and IR64-Sub1) that facilitates further downstream investigation, and (b) compare the whole genome profile of Ciherang-Sub1 with its parental genome sequences using whole-genome shotgun sequencing and reference-guided de novo genome assembly.

## 2. Results and Discussion

### 2.1. Genome Assembly

**Figure 1:**
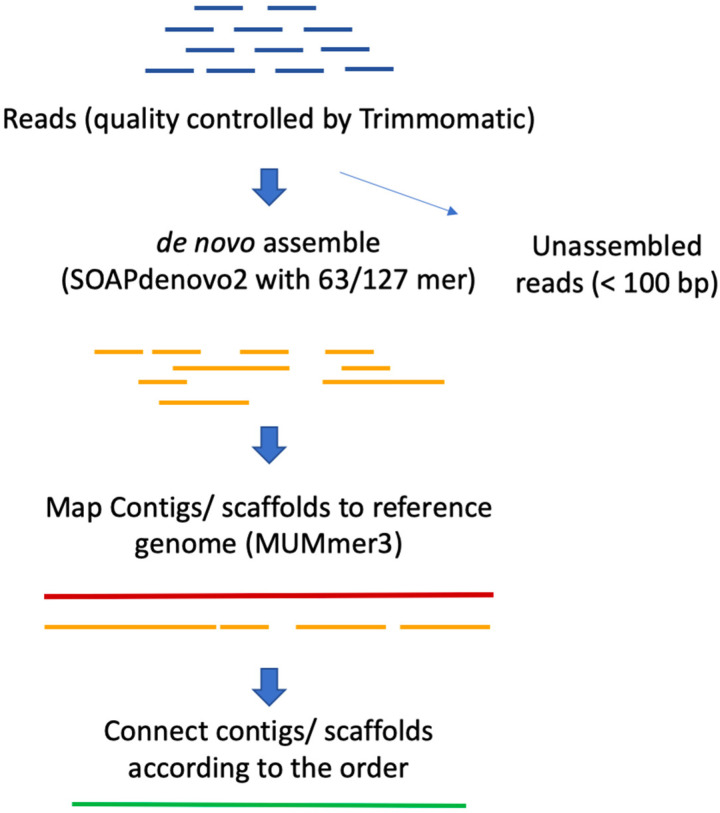
Reference-guided de novo assembly pipeline. The blue, orange, red, and green lines denote raw sequences, clean sequences, the reference sequence, and ordered-contigs/scaffolds, respectively.

The Illumina sequencing results produced more than 100 million raw reads in each cultivar, of which more than 93% of the raw read-pairs were of sufficient quality for use in the de novo assembly (Table S1). A total of 102,213,313 reads from the Ciherang-Sub1 genome, corresponding to 15,331,996,950 bp, were generated, representing 38X sequencing depth and covering 92.6% of the Nipponbare (*Oryza sativa* L. ssp. *japonica*) reference genome and 91.6% of the Minghui 63 (MH63) (*O. sativa* L. ssp. *indica*) reference genome (Table 1). Reference-guided de novo assembly of the three cultivars was combined using the de novo assembler SOAPdenovo2 with a reference genome alignment (Figure 1). In the first step, reads were independently de novo assembled with two different functions, *63mer* and *127mer*. All the three cultivars had better assembly performances with the *63mer* than *127mer* function (Table S2). The average scaffold length of Ciherang-Sub1, Ciherang, and IR64-Sub1 were 6907, 7456, and 7521 bp with *127mer* and 7819, 8115, and 8321 bp with *63mer*, respectively. The longest scaffolds of Ciherang-Sub1, Ciherang, and IR64-Sub1 were 80,935, 101,859, and 120,411 bp with *127mer* and 212,257, 210,201, and 210,221 bp with *63mer*, respectively. Lastly, the N50 values, which demonstrate the length of the 50th percentile of scaffolds, were also measured. In this case, the N50 values of Ciherang-Sub1, Ciherang, and IR64-Sub1 were 11,298, 12,635, and 12,524 bp with *127mer*, respectively, which doubled using the *63mer* function to 25,138, 25,390, and 26,139 bp, respectively. Therefore, the scaffolds assembled with *63mer* were used in the next step.

All scaffolds were aligned to the Nipponbare reference genome and the Minghui 63 (MH63) reference genome separately with the *nucmer* function. The scaffolds were then connected based on their orders to become a complete genome. The results showed that Ciherang-Sub1, Ciherang, and IR64-Sub1 had genome sizes of 345.4, 343.7, and 344.7 Mb, which correspond to 92.2–92.6% coverage on the Nipponbare reference genome (Table 1). For alignment based on the MH63 reference genome, the three cultivars had genome sizes of 353.9–354.9 Mb, which correspond to 91.3–91.6% of the MH63 genome.

### 2.2. Validation of Three Assembled Genomes

The qualities of the three genome assemblies were validated by the position of five known genes, which included *GW5* (*Os05g0187500*), *SD1* (*Os01g0883800*), *Sub1A* (DQ011598), *Sub1B* (*Os09g0287000*), and *Sub1C* (*Os09g0286600*). Results showed that the positions of the best BLAST results, with the highest similarity and coverage (top match, thereafter) of *SD1* and *GW5*, were correct in all three cultivars and aligned well to both Nipponbare (Table 2) and MH63 (Table 3). Previous research revealed that the physical distance between *Sub1A* and *Sub1C* is around 61 kb in FR13A, and the physical distance between *Sub1B* and *Sub1C* is around 15.5 kb in Nipponbare [4]. Our results showed that the top matches of *Sub1B* and *Sub1C* for Ciherang-Sub1 and IR64-Sub1 have similar physical distances with the Nipponbare reference genome. The physical distances between *Sub1B* and *Sub1C* in both Ciherang-Sub1 and IR64-Sub1 are 17.0 and 17.3 kb, respectively. However, for Ciherang, the top match of *Sub1B* is located on chromosome 1, while the top match of *Sub1C* is on chromosome 4. Previous research has indicated that the *Sub1A* gene is absent in *japonica* rice, including Nipponbare, and some other *Oryza* species such as *O. rhizomatis* and *O. eichingeri* [4,15]. Due to this variation in gene presence, *Sub1A* does not have a position in the Nipponbare reference genome. However, we reported the *Sub1A* positions in the three genomes using the MH63 reference genome (Table 3).

Table 3 summarizes the position of five genes in the three genomes based on the MH63 reference. The top match of *SD1* and *GW5* for the three cultivars are located in similar positions on chromosomes 1 and 5, respectively. For *Sub1B*, the top matches for Ciherang-Sub1 and IR64-Sub1 have similar positions with the MH63 reference, both located on chromosome 9. For Ciherang, however, the top match of *Sub1B* is positioned on chromosome 12. For *Sub1C*, the top match for IR64-Sub1 has a similar position with MH63. However, the top matches are on chromosome 10 for Ciherang-Sub1 and chromosome 8 for Ciherang. Interestingly, the top match of *Sub1A* in MH63 itself is on chromosome 6, while for the three cultivars, the top match for *Sub1A* is on chromosome 11.

The results showed that the top match of some *Sub1* genes could have different chromosomal locations in the assembled genome than chromosome 9 reported for FR13A, the donor of *Sub1* [4]. *Sub1B* and *Sub1C* are on the short arm of chromosome 9 in Ciherang-Sub1 and IR64-Sub1 in the Nipponbare reference genome (Table 2). This result reveals that Ciherang-Sub1 and IR64-Sub1 have high similarities in the *Sub1B* and *Sub1C* regions. Marker-assisted breeding depends on either shared relatedness due to recent co-ancestry or linkage disequilibrium (LD) between DNA markers and the genetic variants associated with the phenotypic traits [32]. Indeed, Ciherang-Sub1 was derived from Ciherang and IR64-Sub1, and IR64-Sub1 was the Sub1 donor. When Sub1 was introgressed into Ciherang, it included *Sub1A*, *Sub1B*, and *Sub1C*. Therefore, Ciherang-Sub1 not only has *Sub1A* from IR64-Sub1 but also *Sub1B* and *Sub1C* [8]. The positioning of the top matches of *Sub1B* and *Sub1C* on different chromosomes in Ciherang might be due to the scaffold size that is relatively shorter compared to those of Ciherang-Sub1 and IR64-Sub1; therefore, the top matches may have recognized an ERF-VII related to *Sub1B* or *Sub1C* on different chromosomes. 

*Sub1B* and *Sub1C* are located on the short arm of chromosome 9 in IR64-Sub1 using the MH63 reference genome. *Sub1B* is also positioned on the short arm of chromosome 9 in Ciherang-Sub1; however, *Sub1C* is located on chromosome 10. On the other hand, Ciherang has the top match for *Sub1B* on chromosome 12 and *Sub1C* on chromosome 8 (Table 3). The *Sub1A* gene was not annotated in the MH63 genome [33]. Therefore, we searched for *Sub1A* in MH63. The top match of the complete sequence of *Sub1A* was found at the distal end of chromosome 6 in MH63 and on chromosome 11 in the three cultivars (Table 3). Previous research has indicated that some *indica* varieties might also lack the *Sub1A* locus, such as IR24, Swarna, IR50, and Habiganj Aman [4]. The unexpected location of *Sub1A* in the MH63 genome and its dissociation from *Sub1B* and *Sub1C* could be due to variations in genetic background or an assembling error in MH63. Hence, for our purpose, we used Nipponbare as a reference genome in the variant calling process even though all three cultivars belong to the *indica* rice group.

### 2.3. Genome Profile of Ciherang-Sub1

The genome profile of the three cultivars was examined to identify and characterize SNPs. A total of 590,664 SNPs were detected among Ciherang-Sub1, Ciherang, and IR64-Sub1. Based on the assessment of SNPs, 515,698 (87%) were a Ciherang-like genotype, 65,426 (11%) were an IR64-Sub1-like genotype, and 9540 (2%) were neither Ciherang- nor IR64-Sub1-like (Table 4). We identified a total of 78,190 SNPs on chromosome 8, of which 21,833 SNPs were located within the *qSub8.1* physical interval that varied from 12.4 to 24.1 Mb.

**Figure 2:**
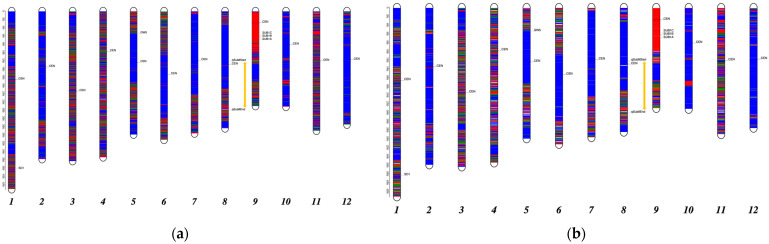
Genome profile of Ciherang-Sub1 with two window sizes: (**a**) 7453 SNPs based on a 50 kb window; (**b**) 3724 SNPs based on a 100 kb window. The blue and red colors represent the Ciherang-like genotype and IR64-Sub1-like genotype; the green color indicates neither Ciherang nor IR64-Sub1. The physical map position (Mb) is shown on the far left. CEN indicates the approximate centromere region according to the Nipponbare reference genome; the orange vertical line indicates the *qSub8.1* QTL region; the positions of *Sub1A*, *Sub1B*, *Sub1C*, *SD1*, and *GW5* are shown. *SUB1A* is represented by a *Sub1* flanking SSR marker, RM8300 (since *Sub1A* is absent in the Nipponbarre genome).

SNPs may cluster on certain chromosome regions (i.e., not evenly distributed along chromosomes), which have important implications for genetic mapping and crop improvement [34]. Therefore, we further examined the genome profile of Ciherang-Sub1 by grouping SNPs into blocks (windows) every 50,000 bp (50 kb) or 100 kb based on their physical positions (Figure S1). A total of 7453 SNPs were identified using a window size of 50 kb, of which 4406 (59%) SNPs were Ciherang-alike and 1791 (24%) SNPs were in the IR64-Sub1-like genotype. The remaining 1256 (17%) SNPs were neither Ciherang- nor IR64-Sub1-like (Table 4). Using a 100kb window size, we identified 3724 SNPs that consisted of 2383 (63%) SNPs that were Ciherang-like, 832 (22%) SNPs that were IR64-Sub1-like, and 564 (15%) SNPs that were neither Ciherang- nor IR64-Sub1-like. In summary, Ciherang-Sub1 has an estimated 59–63% genetic background from Ciherang, 22–24% from IR64-Sub1, and 15–17% from unknown sources. To better visualize the genome profile of Ciherang-Sub1, SNPs from 50 and 100 kb windows were plotted according to their physical position with different colors that represented the genetic backgrounds (Figure 2).

The 50 kb window and 100 kb window analyses reveal similar genome profiles, confirming the accuracy of the genome composition (Figure 2). Both graphs illustrated that most parts of chromosomes 2, 5, 6, 7, 8, 10, and 12 of Ciherang-Sub1 originated from Ciherang, whereas chromosomes 1, 3, 4, and 11 were mosaics of homozygous loci from both Ciherang and IR64-Sub1. The recombination rates, especially around the centromeric regions of some chromosomes (e.g., Chrs 2, 5–8, 10), were very low. Different studies have reported that the distribution of recombination events in diverse crops is often skewed toward the distal ends of the chromosomes, with little to no crossovers near the centromere [35]. This includes chromosome 6A in bread wheat [36] and chromosome 9A in peanut [37]. The genomic landscape could be influenced by several factors, including external processes such as gene flow, background selection, and divergent selection. Background selection in the genomic regions with a reduced recombination rate and share relatedness can generate peaks of relative differentiation, along with gene flow. Additionally, the inherent properties of the genome, such as gene density, recombination rate variation, and mutation rate variation, also play significant roles [38]. Although the majority of the Ciherang-Sub1 genome originated from the recurrent Ciherang parent, almost the entire short arm of chromosome 9 originated from IR64-Sub1, which agrees with the history of Ciherang-Sub1 breeding. Ciherang-Sub1 was developed after just one generation of backcrossing (BC_1_F_2_) using 2 Sub1-flanking microsatellite markers (ART5 and RM8300) for foreground selection and 27 SSR markers for background selection [8]. Although the use of molecular markers for background selection facilitates the recovery of the recurrent parent genome with maximum homozygosity and minimal introgression from the donor parents, at least three generations of backcrossing are required for optimal results [39].

Our analysis found other chromosomal regions that are mosaics of both parents. This may partly be due to recombination events but may also be partly due to the impurity of Ciherang itself since this variety was released about two decades ago [40]. There could be some slight differences between the Ciherang that we sequenced and the Ciherang that was used as the parent of Ciherang-Sub1, which could be due to seed admixture and/or pollen contamination during seed multiplication. In the absence of genotyping quality control methods, such issues are common across multiple species regardless of the mating system, which varied from 3% to 28% [41].

### 2.4. Dissection of qSub8.1 Region

**Figure 3:**
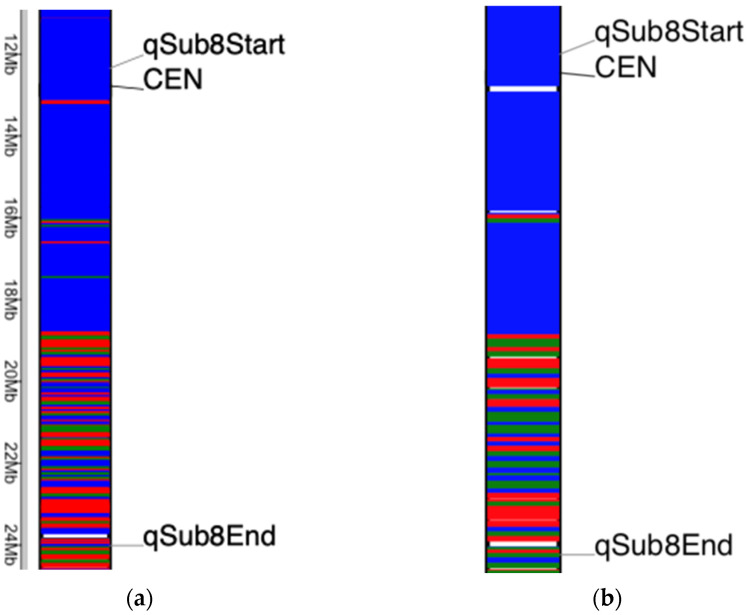
Zoom-in version of the *qSub8.1* region using (**a**) a 50 kb window and (**b**) a 100 kb window size. The blue, red, and green colors represent the Ciherang-like genotype, the IR64-Sub1-like genotype, and neither of them. The white space indicates that the SNP information is missing in the 50 or 100 kb window. The physical map position (Mb) is shown on the left. This QTL region spans between LOC_Os08g20660 and LOC_Os08g38020.

The *qSub8.1* physical interval in the Nipponbare genome varied from 12.4 to 24.1 Mb on chromosome 8 [13]. As shown in Figure 3, most parts of the upper portion of the *qSub8.1* region in the Ciherang-Sub1 originated mainly from the Ciherang background, but the lower portion was inherited from both Ciherang andIR64-Sub1 plus other unknown sources, as described above. The structures profiled with a 50 kb window size and a 100 kb window size were slightly different, which could be due to the noise of the smaller window size. The ideal window size of adjacent markers balances noise reduction with signal identification and maximizing power, which depends on the extent of the linkage disequilibrium (LD), genome size, marker density, and recombination frequency. For example, in common bean, LD levels were found to be higher within the Mesoamerican gene pool and decay more rapidly within the Andean gene pool. The recombination rate across the genome was 2.13 cM/Mb, but it was highly suppressed around the centromeres [34]. The extent of LD varies in rice, and the average LD is about 75 kb in the *indica* subgroup and longer in the *japonica* subgroup [42]. Hence, we selected the two windows of 50 and 100 kb for this study. Based on our analysis, the favorable allele for *qSub8.1* likely originated from IR64-Sub1, but it could also be originated either from a susceptible Ciherang parent or an unknown source due to seed admixture, pollen contamination, or other causes described above. Susceptible parents have previously been reported as the origin of favorable alleles, which includes a major-effect QTL (*qtl12.1*) associated with grain yield under severe drought stress in rice [43]. 

The SNPs identified in the present study will enable us to develop suitable DNA markers and identify the candidate gene(s) underlying *qSub8.1* for further functional characterization. Additionally, transcriptomic studies have been performed to help elucidate the candidate genes underlying the QTL (Bailey-Serres, unpublished data). As described in the introduction section, *qSub8.1* was discovered by using a RIL population derived from the cross between Ciherang-Sub1 that harbored the *SUB1* gene and the submergence and stagnant flooding-tolerant IR10F365 [20]. However, because of complications in importing seeds of IR10F365 (a breeding line), we did not include this parent in the present study, which restricted our ability to identify polymorphic SNPs between IR10F365 and Ciherang-Sub1 around *qSub8.1* that would be useful to narrow the large confidence interval and design breeder-friendly markers that can be used for MAS in the future. Hence, further studies are needed to characterize *qSub8.1* in detail, including fine mapping to narrow its physical interval, validate its effect across different genetic backgrounds, determine/verify the exact parental origin of the favorable allele, and identify polymorphic SNPs within the *qSub8.1* physical interval.

## 3. Materials and Methods

### 3.1. Plant Materials

Ciherang-Sub1, which carries the novel submergence-tolerant QTL *qSub8.1* [20], was derived through marker-assisted backcrossing from a cross between the submergence-susceptible Ciherang as a recurrent parent and the submergence-tolerant IR64-Sub1 as a donor parent [8]. To characterize *qSub8.1*, we used Ciherang-Sub1, Ciherang, and IR64-Sub1 for the whole genome sequencing and assembly. The seeds of Ciherang-Sub1, Ciherang, and IR64-Sub1 were imported from the International Rice Research Institute (IRRI), Philippines, for the experiment and were planted in a quarantined greenhouse in the Texas A&M AgriLife Research Center at Beaumont, Texas, in 2017 for seed multiplication to conduct the research experiment.

### 3.2. Whole Genome Sequencing and Assembly

DNA was collected from healthy leaves of the three cultivars and stored in a −80 °C freezer before extraction. Leaves were ground by mortar and pastel with liquid nitrogen. A QIAGEN DNeasy Plant Mini Kit was used to extract genomic DNA. The quality of the DNA was checked using an Agilent 2100 Bioanalyzer, and the libraries were prepared following the Illumina protocol at the Texas A&M AgriLife Genomics and Bioinformatics Service (TxGen). Whole-genome shotgun sequencing of the three rice genomes was performed using the Illumina HiSeq4000 platform to provide at least 80–100 million reads per sample, with 150 bp pair-ends. The quality of raw reads was checked using Trimmomatic version 0.36 [44] with the PE–Phred 33 command as follows: (1) raw sequencing reads were trimmed to remove adaptors; (2) low-quality bases with a quality score less than 20 on the ends and tails of reads were removed; (3) reads were scanned with a 5 bp sliding window, and those with an average quality of sequences per bp below 20 were removed; (4) reads of less than 25 bases long were dropped; and (5) reads without correspondence read pairs were dropped. We used SOAPdenovo2 version r240 [45] to perform the de novo genome assembly with both *-63mer* and *-127mer* commands. The first command is suitable for assembly with k-mer values less than 63 bp, while the latter one works for k-mer values less than 127 bp. MUMmer version 3.23 [46] was used for aligning de novo scaffolds and contigs to the *O. sativa* L. spp. *japonica* reference genome (IRGSP-1.0) and the *O. sativa* L. spp. *indica* reference genome. The *indica* rice cultivar MH63 version 2 (MH63RS2) reference genome was downloaded from the Rice Information GateWay [47]. Following this, three genomes were constructed by re-orienting and connecting all the scaffolds and contigs according to their order.

### 3.3. Variants Calling

The *O. sativa* L. spp. *Japonica* reference genome (IRGSP-1.0, https://rapdb.dna.affrc.go.jp/download/irgsp1.html; accessed on 15 December 2019) was used as a reference genome in the variants calling. Reads were sorted by individual sample and aligned to the reference genome using Bowtie2 v2.2.9 [48] with the default parameters for end-to-end mode. The likelihood of each genotype was computed using the *samtools mpileup* function in SAMtools v0.1.19 [49], and the actual variant calling was performed using the *bcftools -bvcg* function. SNPs were identified when there were polymorphisms between the three cultivars, and SNPs were filtered with a minimum quality score of 30. To generate the genome structure graph, SNPs of Ciherang-Sub1 were compiled into two different window sizes, (blocks) of 50 and 100 kb, and the dominant SNP types (Ciherang-like genotype or IR64-Sub1-like genotype) within the block were used to represent each block (Figure S1).

## 4. Conclusions

This study demonstrates the potential of whole genomic sequence and genomic information in crop improvement. The results showed that the Ciherang-Sub1 genome is composed of 59–63% Ciherang, 22–24% of IR64-Sub1, and 15–17% of unknown sources. The genome profile derived from SNP information provided a more detailed picture of the previous Ciherang-Sub1 marker-assisted breeding. High-resolution SNP markers allow us to better understand *qSub8.1* chromosome 8. The SNPs identified in the current study can be used to assist in analyzing the candidate genes underlying *qSub8.1* and to develop DNA markers for future breeding efforts to further improve the level of submergence tolerance in rice. However, further studies are needed to fine-map the QTL, design breeder-friendly SNPs for MAS in the future, validate its effect across different genetic backgrounds, determine/verify the exact parental origin of the favorable allele, and conduct gene expression analysis on the QTL region.

## Figures and Tables

**Table 1:** General sequencing statistics and summary of assembly.

				Genome Size (bp)	
Variety	Number of Reads	Total Read Length (bp)	Sequencing Depth (X)	Nipponbare Reference *	MH63 Reference *
Ciherang-Sub1	102,213,313	15,331,996,950	38	345,442,284 (92.6%)	354,934,762 (91.6%)
Ciherang	115,172,273	17,275,840,950	43	343,737,849 (92.2%)	353,859,778 (91.3%)
IR64-Sub1	114,085,140	17,112,771,000	43	344,678,967 (92.4%)	353,859,751 (91.3%)

* The Nipponbare and MH63 reference genome sizes are 373 and 387 Mb, respectively.

**Table 2:** Summary of the position of the top match of known genes in the three genomes based on the Nipponbare reference genome.

Gene	Position of the Top Match in Each Genome Assembly (bp)			
	Nipponbare	Ciherang-Sub1	Ciherang	IR64-Sub1
*SD1* (*Os01g0883800*)	chr01:38382385-38385469	chr01:42499233-42501392	chr01:43076922-43079161	chr01:42595591-42597830
*GW5* (*Os05g0187500*) *Sub1A* (DQ011598)	chr05:5365122-5366701 absent ^1^	chr05:4832476-4834024 NA ^2^	chr05:4873155-4874734 NA	chr05:5262475-5264054 NA
*Sub1B* (*Os09g0287000*)	chr09:6404482-6406039	chr09:5266747-5267932	chr1:4258518-4259713	chr09:4932683-4933868
*Sub1C* (*Os09g0286600*)	chr09: 6387891-6389789	chr09: 5250965-5252048	chr4: 13931868-13933764	chr09: 4916471-4917910

^1^ Gene not present in the Nipponbare genome. ^2^ NA, not applied.

**Table 3:** Summary of the position of the top match of known genes in the three genomes based on the MH63 reference genome.

Gene	Position of the Top Match in Each Genome Assembly (bp)			
	MH63	Ciherang-Sub1	Ciherang	IR64-Sub1
*SD1* (*OsMH_01G0636900*)	chr01:39643093-39644426	chr01:42732034-42734193	chr01:42311555-42313794	chr01:42124122-42126361
*GW5* (*OsMH_05G0081900*)	chr05:5428533-5430112	chr05:5278241-5279789	chr05:5178118-5179697	chr05:4864637-4866216
*Sub1A* (DQ011598)	chr06:22422489-22419199	chr11:17912307-17916149	chr11:8553735-8556945	chr11:18314723-18318566
*Sub1B* (*OsMH_09G0114700*)	chr09: 7179132-7180335	chr09: 4262487-4264027	chr12: 7911412-7912963	chr09: 5209175-5210715
*Sub1C* (No annotation)	chr09: 7162325-7164223	chr10: 10781009-10782407	chr8: 20306150-20308046	chr09: 5193229-5195103

**Table 4:** Genome profile of Ciherang-Sub1 with different window sizes.

	All SNPs	50 kb Window Size	100 kb Window Size
Ciherang-like genotype	515,698 (87%)	4406 (59%)	2328 (63%)
IR64-Sub1-like genotype	65,426 (11%)	1791 (24%)	832 (22%)
Other	9540 (2%)	1256 (17%)	564 (15%)
Total SNP number	590,664	7453	3724

## Data Availability

Data are available as Supplementary Materials.

## Supplementary Materials

The following are available online at https://www.mdpi.com/article/10.3390/plants10122740/s1, Figure S1: The illustration of examined SNP blocks using a 50 or 100 kb window size, Table S1: Raw sequence outputs of three rice genomes, Table S2: De novo assembly of three rice genomes using different assembly functions.
