# Supplementary material for: Reference-Guided De Novo Genome Assembly to Dissect a QTL Region for Submergence Tolerance Derived from Ciherang-Sub1
Source: Plants (Basel). 2021 Dec 13;10(12):2740. doi: 10.3390/plants10122740 (PMC8703405; doi:10.3390/plants10122740)
Supplement: Supplementary file 1 [file plants-10-02740-s001.zip › plants-1470037-supplementary.pdf]

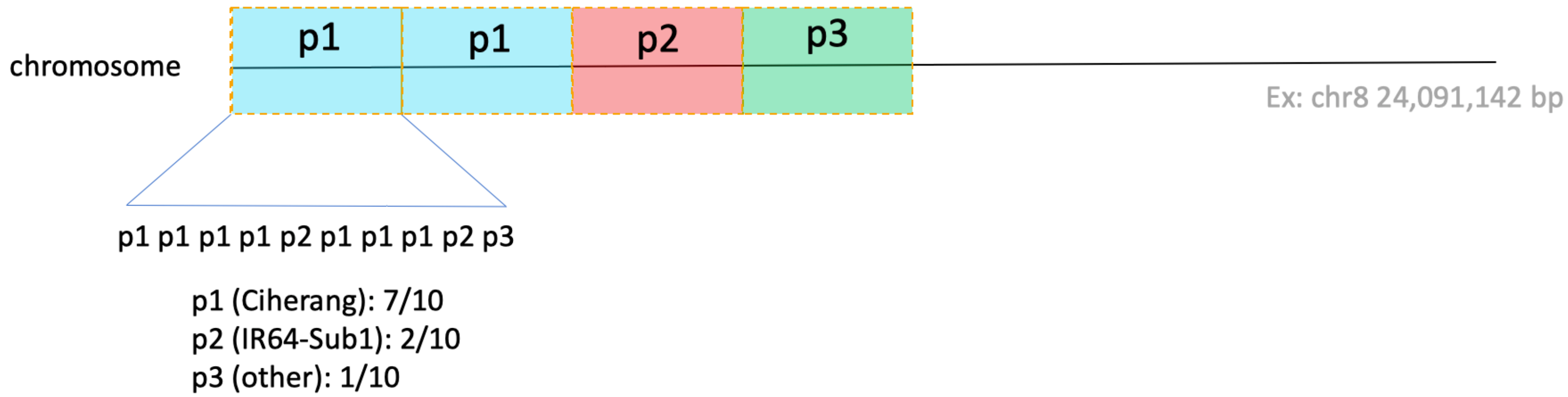

Figure S1. The illustration of examined SNP blocks using 50kb or 100kb window size.

Table S1. Raw sequence outputs of three rice genomes.

Variety	Number of raw read pairs	Number of read pairs after trimming
Ciherang-Sub1	107,957,981	102,213,313 (94.68%)
Ciherang	122,304,187	115,172,273 (94.17%)
IR64-Sub1	122,140,197	114,085,140 (93.41%)

Table S2. *De novo* assembly of three rice genomes using different assembly functions.

	127mer			63mer		
	Ciherang-Sub1	Ciherang	IR64-Sub1	Ciherang-Sub1	Ciherang	IR64-Sub1
Number of scaffold	43595	40208	39881	40341	38595	37731
Average length (bp)	6907	7456	7521	7819	8115	8321
Length of longest scaffold (bp)	80935	101859	120411	212257	210201	210221
N50 (bp)	11298	12635	12524	25138	25390	26139
N90 (bp)	1372	1515	1595	514	354	468
